# Gleanings from Our Exchanges

**Published:** 1873-08-15

**Authors:** 


### Gleanings from Our Exchanges.

Delivery of the Head in Breech Presentation.—Dr. Hannibal Langdon, of Ligonier, Ind., in a very interesting communication addressed to the editors of the *American Practitioner*, advises the following practice in the delivery of the head in breech presentations, and states that he has proved its value in numerous cases:

"Breech presentations occur once in sixty times, and the mortality to children is about one in three and a half. The dangers to the child are from compression of the cord, detachment of placenta, inertia of the uterus after the body is born, etc. The remedy is *rapid* delivery. But you may not have the advised forceps at hand; you have, however, your fingers, which can be more promptly and successfully used than any instruments. This is the method of proceeding: The infant's body is delivered with its back superior, the patient lying upon her back. First draw the cord down a little way; then if the head has passed the superior strait, the face is in the hollow of the sacrum; if not, bring it down, according to the usual rules, as rapidly as possible. Then introduce the index-finger of one hand into the mouth of the child, drawing the chin down, at the same time with the fingers of the other hand push the occiput up, thus securing perfect flexion. This accomplished, the face of the child will present at the vulva; and immediately withdraw the finger from the infant's mouth, and pass two fingers into the rectum of the patient, and you readily reach the vertex and use these fingers as a lever, lifting *upward* and *outward*, while a similar movement is communicated to the body of the child with the other hand placed below it. If you are on the patient's right side, your index and middle fingers of the right hand will be against the vertex of the child; if upon your left, those of your left hand. If unfortunately you have failed to deliver the body with the back superior, and you have the face toward the pubes, the same general steps are necessary, save that the finger of your right or left hand, as the case may be, should be *kept* in the child's mouth while the upward and outward movement is made with the fingers on the vertex. This method of delivery is applicable to all cases where the body of the child is born first. By it the head can be delivered in less time than required for the application of forceps, and it is much safer for the mother at least. Pursuing it, I have never lost a child in breech presentation, or in podalic version."

Therapeutical Effect of Liquor Sodæ Chloratæ.—Dr. Cooper advocates the use of Labarraque's solution in relaxed states of the uterine and periuterine tissues, and thinks that it gives tone to the weakened utero-sacral ligaments, increases vaginal contractions, removes the bearing down and tendency to prolapse, diminishes congestion of the neck of the womb, thereby lessening very considerably leucorrhœal and menorrhagic discharges; and these effects are followed by the entire subsidence of sympathetic disturbances, the sacral, rectal, and ovarian distress, the hypochondrial tumefaction, the gastric and precordial sinking, the sub-mammary stitch, and chest pains and headache. He recommends two or three drops of the ordinary commercial solution to be placed in half a tumblerful of water, and directs that a dessert-spoonful of this be taken occasionally. Should disagreeable symptoms be produced by the medicine—*e. g.*, rapid emaciation and influenza-like debility, which is its most uniformly unfavorable symptoms—it should be discontinued for some time. As an ingredient of vaginal douches in ulceration of the neck of the womb, hypochlorite of soda gives great relief.—*Dub. Jour. of Med. Sci., April.* —*Medical Record.*

Clinical Illustrations of the Value of Phosphorus in Certain Forms of Disease of the Nervous System.—Under this heading, Dr. W. H. Broadbent, of London, relates cases of epileptiform vertigo, severe neuralgia with gastric complications, nervous breakdown from overwork, and atonic dyspepsia. He thinks the failure on the part of the phosphorus to answer the expectations of those who thought a few years since they had in it a powerful remedy in this class of affections, is to be explained by the difficulty of administering it without destruction ; or, at any rate, impairment of its activity by oxidation. He says that instead of giving it as formerly, dissolved in almond or olive oil, and suspended in mucilage, in which form oxidation takes place slowly, but which forms a nauseating mixture, he has administered it dissolved in oil or lard and inclosed in a gelatine capsule; the dose is about $\frac{1}{30}$ of a grain, and it may be taken two or three times a day—always after food. When dissolved in cod-liver oil phosphorus appears to undergo oxidation more rapidly than in any other medium by which it has been administered; and in the form of pill its deterioration is but little less rapid. Dr. Broadbent thinks it as absurd to substitute phosphoric acid for phosphorus as it would be to expect sulphuric acid to give us the therapeutical effects of sulphur.—*The Practitioner.—Med. Record.*

Phosphorus Mixture.—A very pleasant preparation of phosphorus, in the fluid form, is: Dissolve a grain of phosphorus in six drachms of chloroform, and add two ounces of good glycerine, and shake well. The glycerine forms a permanent union with chloroform, which dilution with water does not separate. Dose, a teaspoonful three times a day.

Hypophosphorus acid has not received that attention its merits entitle it to. As a remedial agent of the phosphorus family, it is one of the most reliable. The hypophosphorus acid, in solution, enters the economy in combination with some of the fatty acids and glycerine combined, and forms a true nerve food and restorative. For children it is of decided advantage. The usual dose is from ten drops to ʒ ss., with an equal quantity of glycerine in half to an ounce of some aromatic water.—*Medical Archives.*

Boils, their Cause and Cure.—Dr. J. D. McGaughey, in *Medical Times* (March 8), gives some good reasons for thinking that the probable cause of boils is a temporary increase of the white blood corpuscles. For their cure he recommends the use of quinine in doses sufficient to affect the head, and continued till recovery. Arsenic he thinks the next best remedy. This view is further supported by the observations of Binz. He found that quinine kills the white blood corpuscles, and when given by the mouth the quantity of white blood corpuscles in the blood was diminished.—*South. Med. Record.*

Treatment of Chloroform Narcosis by Ice in the Rectum.—Dr. Baillee thinks there is no readier or more effectual mode of combating the narcosis of chloroform than that of introducing a small piece of ice into the rectum. But little force is required to pass it through the anus; it melts immediately, and causes by its presence a deep reflex inspiration, which leads to respiratory movement and re-establishment of the cardiac function. The method is also recommended to be pursued in the treatment of apparent death in the newly-born.—*Bul. Gen. de Therap.*, *April* 15.—*Med. Record.*

Dr. Hanot recently reported to the *Societe de Biologie* in Paris a case in which uræmia, supervening on general paralysis, was diagnosed by the symptom pointed out by Bourneville—a rapid and general decline of temperature.

The Regular Profession in Philadelphia. — The *Philadelphia Medical Register* states that there are in that city 699 regular physicians. Of these, 50 are on the retired list.

Bromide of Potassium.—The subjoined conclusions concerning bromide of potassium are noted by M. Gonzales Echeverria, of New York (*Philadelphia Medical Times*), based on clinical facts: The remedy operates actively in a paralyzing manner on the excito-motor power of the sympathetic and spinal nervous system, and produces cerebral congestion. It is not a specific for epilepsy, but may contribute most efficiently to arrest the fits, by suspending reflex excitability of the spinal cord in a more uniform and active manner than any of its kindred salts. The benefits of the bromide of potassium increase and are better secured by administering it combined with conium, ergot, arsenic, and strychnia. Sixty grains is the average daily amount of bromide of potassium required in ordinary cases of epilepsy; but it is necessary to keep epileptics, at different intervals during the progress of their attacks, under the full physiological effects of the bromide, to suspend it until the bromism is dispelled, and then to resume the salt again in ordinary doses. It alone exerts little or no effect on the attacks of *petit mal*, unless associated with large doses of ergotine (gr. vi. to gr. xvi.) and conium. The remedy should never be discontinued suddenly, even when the epileptic fits have not recurred in a long time, but the dose of the salt should be gradually decreased, and the treatment left off insensibly, when there is every indication of the complete eradication of the epileptic habit. Nothing proves that the efficacy of the bromide of potassium bears any relation to the existence or severity of the bromide eruption. Arsenic prevents or mitigates it, and acts further as a valuable tonic. Coffee is one of the best means of correcting the unpleasant action of full doses of the bromide. When it is taken by the mother in large and prolonged doses, it does not seem to have any noxious influence on the fœtus.—*N. Y. Med. Rec.*

Oxide of Zinc in the Diarrhœa of Infants and Young Children.—Dr. Brakenridge, of Edinburgh, whose experience is very extensive, and who has employed all the remedies in use for infantile diarrhœa, gives the preference to the oxide of zinc. He says: 1. Diarrhœa in these cases arises from a condition of debility and great susceptibility of the nervous centers, which prevent proper secretion from the alimentary tract. 2. It is intimately associated with convulsions and convulsive affections. 3. It is accompanied by congestion of the secreting surface of the digestive passages.

To meet these conditions requires a remedy which is at once tonic, antispasmodic, and astringent. These properties he believes to be united in the oxide of zinc. It is a tonic for the nervous system, just as iron is for the blood. As an antispasmodic and astringent it has already gained a reputation founded on clinical experience. He has employed it in twelve cases, four of them girls and eight of them boys, and varying in age from four months to one and a half years. The form was usually that of the powder, but it was also given in a solution of gum arabic, with a slight addition of glycerine. The general results observed were: 1. That it moderated the diarrhœa quickly. 2. That vomiting stopped. 3. That digestion improved. 4. That intestinal hæmorrhage was frequently arrested. 5. Teething was favored rather than otherwise. 6. That even where no change was made in diet, and the other conditions remained the same, the treatment progressed favorably. 7. When, however, diet and regimen were carefully regulated, success was more rapid and decided.—*Med. Times and Gazette.*

Treatment of Hooping-Cough.—Dr. Charles Kelly, in the *Practitioner* for March, 1873, reports some experiments with belladonna in this disease. It was given in full doses, gtts. x to ʒ ss. of the tincture every two, three or four hours, for four or five days at a time; and the effect seemed to be to lessen the severity and duration of the coughing spells.

Dr. Berry, of Lancaster, is quoted in the same journal as advocating the use of dilute nitric acid, gtts. v. to xv., given in simple syrup every three or four hours. He thinks it not only allayed the cough and spasm, but actually cut short the disease.—*South. Med. Record.*

The First Operation of Lithotripsy in America.—Dr. Alban Goldsmith, the first assistant to Dr. McDowell, in many of his ovariotomies, made the first operation of lithotripsy ever performed in Kentucky or in the United States. It occurred in 1829.

The First English Obstetrical Writer.—The first English midwife who appeared as an obstetrical writer was Mrs. Jane Sharp, of London, whose work was published in 1671, under the name of the "Midwives' Book"—a duodecimo of 418 pages.

Excision of the Ankle Joint.—The first English surgeon who performed the difficult operation of excision of the ankle joint was Henry Hancock, senior surgeon of Charing Cross Hospital, and President of the Royal College of Surgeons, London.

